# Diagnostic and prognostic value of circRNAs expression in head and neck squamous cell carcinoma: A meta‐analysis

**DOI:** 10.1002/jcla.24496

**Published:** 2022-05-20

**Authors:** Huajun Feng, Dingting Wang, Jinping Liu, Longfei Zou, Shengen Xu, Zhuoping Liang, Gang Qin

**Affiliations:** ^1^ Department of Otolaryngology Head and Neck Surgery The Affiliated Hospital of Southwest Medical University Luzhou China; ^2^ Department of Orthopedic Surgery The Affiliated Hospital of Southwest Medical University Luzhou China

**Keywords:** biomarker, circular RNA, head and neck squamous cell carcinoma, diagnosis, prognosis

## Abstract

**Background:**

Circular RNAs (circRNAs) have been found to have potential biological applications against tumors in humans. This study aimed to evaluate the diagnostic, prognostic, and clinicopathological value of circRNAs in head and neck squamous cell carcinoma (HNSCC).

**Methods:**

The PubMed, Web of Science, EMBASE, and the Cochrane Library were comprehensively searched for the relevant studies before October 20, 2021. Statistical analysis was performed based on STATA 15.0, Meta‐DiSc 1.4, and RevMan 5.3 software.

**Results:**

A total of 55 reports regarding 56 kinds of circRNA were studied in this meta‐analysis, including 23, 38, and 26 articles on diagnosis, prognosis, and clinicopathological features, respectively. The pooled sensitivity, specificity, and area under the curve (AUC) of the summary receiver‐operating characteristic curve (SROC) were 0.78, 0.84, and 0.87, respectively. Besides, the upregulation of oncogenic circRNAs was significantly associated with poorer overall survival (OS) (HR=2.25, *p* < 0.05) and disease‐free interval (DFS) (HR=1.92, *p* < 0.05). In contrast, the elevated expression of tumor suppressor circRNAs was associated with a favorable prognosis (HR=0.50, *p* < 0.05). In addition, the high expression of oncogenic circRNAs was associated with the tumor size (OR=3.59, *p* < 0.05), degree of differentiation (OR=1.89, *p* < 0.05), TNM stage (OR=2.35, *p* < 0.05), lymph node metastasis (OR=1.85, *p* < 0.05), and distant metastasis (OR=3.42, *p* < 0.05). Moreover, the expression of tumor suppressor circRNAs was associated with improved clinicopathological features (lymph node metastasis: OR=0.25, *p* < 0.05).

**Conclusions:**

CircRNAs could serve as potential predictive indicators and be useful for the diagnosis, prognosis, and identification of clinicopathological features in HNSCC.

## Graphical abstract

This study aimed to evaluate the diagnostic, prognostic, and clinicopathological value of circRNAs in patients with head and neck squamous cell carcinoma (HNSCC). The pooled sensitivity and specificity, diagnostic odds ratio (DOR), and overall area under the curve (AUC) of the summarizing receiver‐operating characteristics (SROC) were determined and used to assess the diagnostic value of circRNAs. In addition, subgroup analyses were performed to explore the main sources of heterogeneity. Pooled hazard ratios (HRs) and 95% confidence intervals (CIs) were calculated to assess the association between circRNAs expression, overall survival (OS), and disease‐free interval (DFS). Pooled odds ratios (ORs) and 95% CIs were used to assess the relationship between circRNAs expression and clinicopathological parameters. (A) Forest plots of sensitivity and specificity for the diagnosis of circRNAs in HNSCC among 31 studies. (B) The AUC (SROC curve) for the diagnosis of circRNAs in HNSCC among 31 studies. (C) Forest plots of the association between the expression of circRNAs and OS in HNSCC. (D) Forest plots of the association between the expression of circRNAs and DFS in HNSCC.

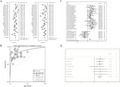

## 1 INTRODUCTION

Head and neck squamous cell carcinoma (HNSCC) is the most common group of head and neck malignancies. Although this group of malignancies originates in different sites at the head and neck, including (1) nasal cavity and sinuses, (2) nasopharynx, (3) hypopharynx, larynx, and trachea, and (4) oral and oropharynx, their pathogenesis, staging system, treatment strategies, and prognosis are similar. Therefore, it is reasonable to group them together as HNSCC^1^. HNSCC is the fifth most common cancer occurring worldwide, with over 600,000 cases reported annually^2^, ^3^. Despite advances in surgery, chemotherapy, immunotherapy, and radiotherapy, the 5 years survival rate of HNSCC patients still remains between 40%–50%^4^. Since the overall survival rate of patients with HNSCC has barely improved over the past few decades, it is critical to identify new molecular markers for the early detection and prognosis and identify new therapeutic targets for HNSCC addressing this dismal clinical situation^5^, ^6^.

Circular RNAs (circRNAs), a new class of endogenous noncoding RNAs, are characterized by a closed‐loop structure formed by covalent bonds between the head and tail, and are usually generated by the exons of precursor mRNAs through reverse splicing^7^, ^8^. CircRNAs may regulate carcinogenesis in different cancers by performing their complex biological functions, i.e., by acting as ceRNA or miRNA sponges, regulating regulatory gene transcription and expression, interacting with RNA‐binding proteins, and translating RNAs into proteins. Because circRNAs are also more stable and conserved than linear RNAs, numerous circRNAs can occur in exosomes, peripheral blood, or tissues^8^, ^9^, ^10^, ^11^. CircRNAs may be suitable for use as novel biomarkers and therapeutic targets for human cancer.

Studies have shown that circRNAs are abnormally expressed in numerous human cancers including esophageal cancer^12^, osteosarcoma^13^, lung cancer^14^, and breast cancer^15^. Simultaneously, several studies have confirmed the role of circRNAs in the proliferation, migration and invasion, apoptosis, angiogenesis, deterioration, and recurrence of human cancer^16^, ^17^, ^18^, ^19^. These results indicate that circRNAs have significant potential for use in human cancer prediction, and prognosis and clinical treatment. CircRNAs can act as both tumor suppressors and oncogenes in HNSCC^20^. Therefore, circRNAs may act as a new biomarker and therapeutic target for the prevention and treatment of HNSCC. However, inconsistent results from existing studies have become an obstacle to the application of circRNAs in clinical practice.

To our knowledge, no meta‐analysis has been performed till date to assess the diagnostic and prognostic value of circRNAs in HNSCC. Therefore, we conducted a systematic and comprehensive meta‐analysis of relevant studies, to explore the significance of circRNAs in the diagnosis and prognosis of HNSCC.

## 2 MATERIALS AND METHODS

### 2.1 Search strategy

This study was performed in accordance with the Preferred Reporting Items for Systematic Review and Meta‐analysis (PRISMA) Checklist^21^. As of October 20, 2021, we conducted a comprehensive search to identify studies that assessed the association of circRNAs with the diagnosis and prognosis or clinicopathological features of HNSCC using 4 electronic databases, i.e., PubMed, Web of Science, EMBASE, and the Cochrane Library database. The following terms were used in databases for report retrieval: (RNA, Circular OR circRNA OR Circular RNA OR ciRNA) AND (cancer OR tumor OR neoplasm OR tumor OR malignant OR metastasis OR carcinoma OR Squamous Cell Carcinoma OR SCC) AND (head and neck OR larynx OR oropharynx OR hypopharynx OR nasopharynx OR oral and cavity OR mouth OR laryngeal OR pharyngeal OR sinus OR sinonasal OR tongue OR NPC OR nasopharyngeal).

### 2.2 Study selection

Studies that met the following criteria are included: (1) cohort or case‐control studies; (2) studies in which HNSCC was histopathologically confirmed; (3) studies that evaluated the association between circRNAs expression, with the diagnosis, prognosis, and clinicopathological features of HNSCC.

The following reports were excluded: (1) studies not related to circRNAs or HNSCC; (2) reviews, case reports, or retracted studies; (3) studies involving animal experiments or cell line experiments; (4) studies lacking sufficient data; (5) studies that were not in English.

### 2.3 Data extraction and quality assessment

Two independent investigators (FHJ and WDT) evaluated the included studies and carefully extracted the data, and if disagreements occurred, a third investigator (LJP) was consulted to reach a consensus. The following data were extracted from the relevant studies: (a) basic characteristics: first author, publication date, country, sample size, sample type, circRNAs name, regulatory characteristic, cancer type, detection method, control type, and follow‐up time; (b) data acquired in diagnostic studies: TP, FP, FN, TN, sensitivity (SEN), specificity (SPE), area under the curve (AUC); (c) data for prognostic studies: hazard ratio (HR) values and 95% confidence interval (CI) of survival outcomes; and (4) clinicopathological features: age, sex, TNM stage, T stage, lymph node metastasis, distant metastasis, tumor size, and degree of differentiation.

The effect of the quality of included studies on diagnosis was assessed according to the Quality Assessment for Studies of Diagnostic Accuracy II (QUADAS II) checklist^22^. Studies on prognosis were rated by the Newcastle‐Ottawa Scale (NOS), as described previously^23^, ^24^. Studies were considered to be of high quality if the QUADAS II score was ≥4 or the NOS score was ≥6.

### 2.4 Statistical analysis

Statistical analysis was performed using Stata 15.0, Revman 5.3, and Meta‐DiSc 1.4 software. The TP, FP, FN, and TN values were calculated to determine the pooled sensitivity, specificity, AUC, negative likelihood ratio (NLR), positive likelihood ratio (PLR), and diagnostic odds ratio (DOR) at the corresponding 95% CI, to evaluate the diagnostic value of circRNAs in HNSCC. The corresponding 95% CI value of the HRs was used to evaluate the relationship between circRNAs and the prognosis of HNSCC patients. The association between circRNAs expression and clinicopathological parameters was assessed using a combination of odds ratios (ORs) with a 95% CI. The threshold effect was evaluated using a Spearman’s correlation coefficient, and values were considered statistically significant if *p* < 0.05. The nonthreshold effect was tested using the Cochran's *Q* test and the I^2^ test, and the level of statistical significance was set as *p* < 0.01 or I^2^ >50%. When there is no heterogeneity between studies, fixed‐effect models can be used to merge data. Otherwise, the random‐effects model is used. The source of heterogeneity was traced using sensitivity analysis and meta‐regression tests. The Deek’s funnel plot asymmetry test for the diagnostic meta‐analysis, *p* < 0.01, was considered statistically significant. And publication bias between studies about prognosis was evaluated using the Begg’s test and Egger’s test, *p* < 0.05 was considered statistically significant.

## 3 RESULT

### 3.1 Search results

**FIGURE 1:**
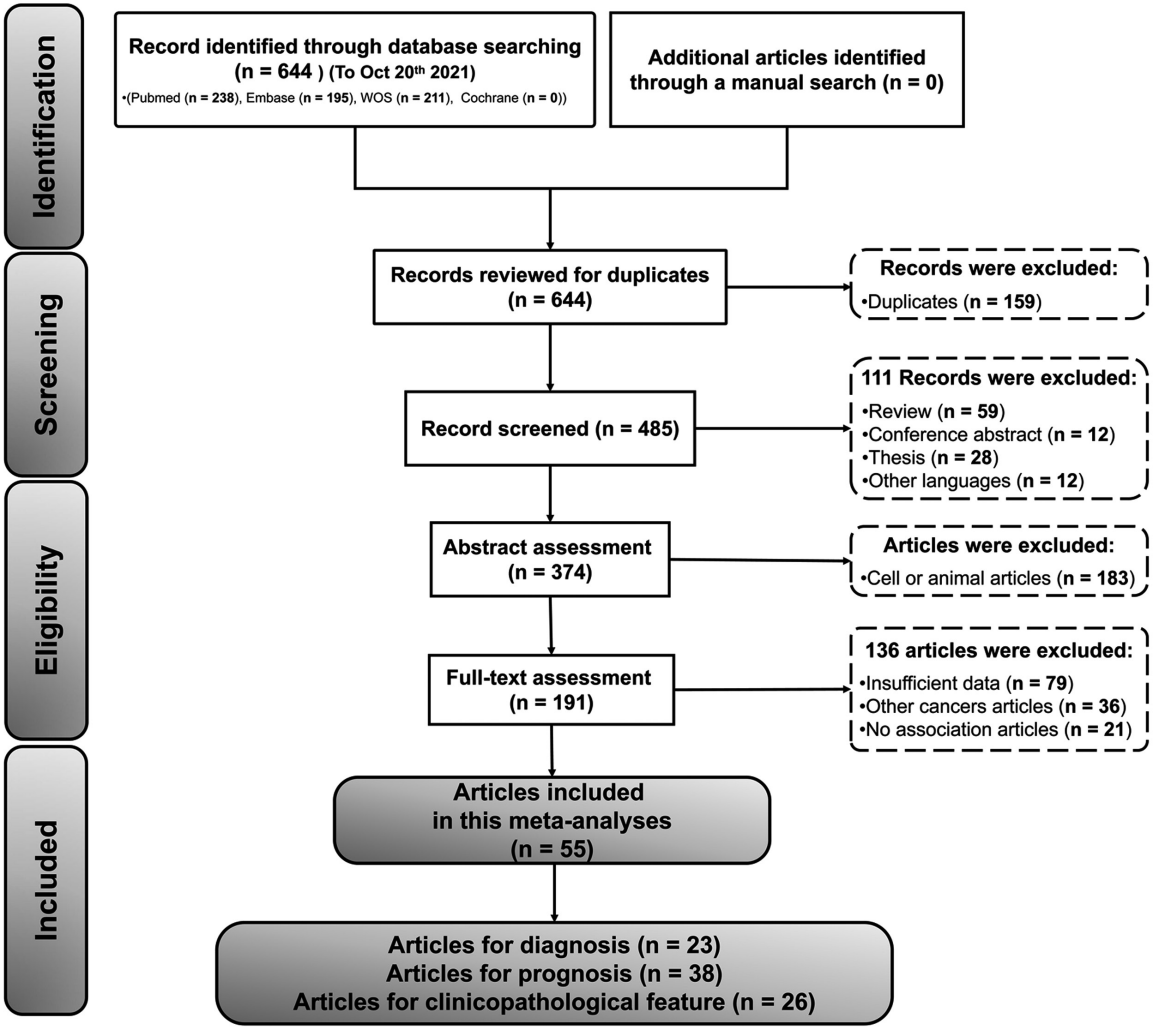
The flow chart of the research selection process

The process for the selection of research articles to be reviewed is shown in Figure 1. A total of 644 potential literatures were initially identified via database searches. After 159 duplicate publications were excluded the titles and abstracts of the remaining 485 articles were assessed. Among these, 294 articles were excluded after reviewing for various reasons, and only 191 articles were reviewed thoroughly. Finally, 55 articles that involved details regarding 56 unique circRNAs and 5,576 HNSCC cases (all cases were reliably diagnosed via histopathological analysis) were included in this meta‐analysis. To be specific, we included 31 diagnostic studies (from 23 articles^25^, ^26^, ^27^, ^28^, ^29^, ^30^, ^31^, ^32^, ^33^, ^34^, ^35^, ^36^, ^37^, ^38^, ^39^, ^40^, ^41^, ^42^, ^43^, ^44^, ^45^, ^46^, ^47^), 38 prognostic studies (from 38 articles^25^, ^26^, ^27^, ^28^, ^29^, ^30^, ^48^, ^49^, ^50^, ^51^, ^52^, ^53^, ^54^, ^55^, ^56^, ^57^, ^58^, ^59^, ^60^, ^61^, ^62^, ^63^, ^64^, ^65^, ^66^, ^67^, ^68^, ^69^, ^70^, ^71^, ^72^, ^73^, ^74^, ^75^, ^76^, ^77^, ^78^, ^79^), and 27 clinical‐pathological feature‐related studies(from 26 articles^28^, ^29^, ^43^, ^47^, ^48^, ^50^, ^51^, ^53^, ^55^, ^56^, ^58^, ^63^, ^64^, ^65^, ^66^, ^67^, ^68^, ^70^, ^71^, ^72^, ^73^, ^74^, ^76^, ^77^, ^78^, ^79^).

### 3.2 Study characteristics and quality assessment

Table 1 and Table 2 show the basic characteristics of the included studies. 55 articles were included, 54 articles were conducted in China and 1 article in Italy. The number of patients in each study had an individual range of 20–292. All studies were published between 2017 and 2021 and were conducted for 20–90 months. As shown in Table 1, the diagnostic meta‐analysis of 31 eligible studies was performed; of these, 23 articles involved reports regarding 26 types of circRNAs. The quality assessment of these 23 articles was performed as shown in Figure S1. The expression of circRNAs in all diagnostic studies was determined by quantitative real‐time polymerase chain reaction (qRT‐PCR) analysis of tissues (*n* = 18), plasma (*n* = 9), serum (*n* = 3), and saliva (*n* = 1). There are a total of 16 upregulated circRNAs and 15 downregulated circRNAs. Tumor types included OSCC (*n* = 15), LSCC (*n* = 8), NPC (*n* = 4), HPSCC (*n* = 2), TSCC (*n* = 1), and HNSCC (*n* = 1). As shown in Table 2, we performed a prognostic meta‐analysis of 38 relevant studies that assessed the association between circRNAs and OS, and 7 studies that assessed the association between circRNAs and DFS. Our prognostic meta‐analysis showed a total of 30 circRNAs upregulated (tumor promoters) and 8 circRNAs downregulated (tumor suppressors) in HNSCC. The expression of circRNAs was calibrated in most studies using qRT‐PCR analysis, except for 3 studies in which the expression was calibrated using in situ hybridization (ISH). Species included serum (*n* = 3) and tumor (*n* = 35) samples. Tumor types included OSCC (*n* = 12), LSCC (*n* = 7), NPC (*n* = 10), HPSCC (*n* = 2), TSCC (*n* = 3), and HNSCC (*n* = 4).

**TABLE 1:** Main characteristics of studies for diagnosis analysis in HNSCC

Study	Year	Country	CircRNAs	Regulation	Sample size		Cancer	Specimen	Method	Diagnostic power			Source of the control group
					Case	Control				SEN	SPE	AUC	
Fan C(a) et al	2019	China	CircMAN1A2	Upregulated	100	121	NPC	Serum	qRT‐PCR	0.81	0.86	0.91	healthy controls
Wang(a) J et al	2020	China	Hsa_circ_0066755	Upregulated	16	19	NPC	Tissue	qRT‐PCR	0.88	0.84	0.90	nasal polyps tissues
Wang(b) J et al	2020	China	Hsa_circ_0066755	Upregulated	86	86	NPC	Plasma	qRT‐PCR	0.86	0.79	0.85	healthy controls
Shuai M et al	2020	China	Hsa_circ_001387	Upregulated	100	100	NPC	Tissue	qRT‐PCR	0.70	0.96	0.92	adjacent normal tissues
Yao Y et al	2020	China	Hsa_circ_0001742	Upregulated	146	146	TSCC	Tissue	qRT‐PCR	0.78	0.81	0.87	adjacent normal tissues
Wang X et al	2020	China	Hsa_circ_103862	Upregulated	62	62	LSCC	Tissue	qRT‐PCR	0.82	0.69	0.81	adjacent normal tissues
Guo Y et al	2020	China	Hsa_circ_0036722	Downregulated	41	41	LSCC	Tissue	qRT‐PCR	0.61	0.95	0.84	adjacent normal tissues
Han J(a) et al	2021	China	Hsa_circ_0019201	Upregulated	20	20	LSCC	Plasma	qRT‐PCR	0.95	0.85	0.93	healthy controls
Han J(b) et al	2021	China	Hsa_circ_0019201	Upregulated	100	100	LSCC	Plasma	qRT‐PCR	0.64	0.95	0.77	healthy controls
Han J(c) et al	2021	China	Hsa_circ_0011773	Upregulated	20	20	LSCC	Plasma	qRT‐PCR	1.00	0.75	0.91	healthy controls
Han J(d) et al	2021	China	Hsa_circ_0011773	Upregulated	100	100	LSCC	Plasma	qRT‐PCR	0.78	0.98	0.86	healthy controls
Han J(e) et al	2021	China	Hsa_circ_0122790	Upregulated	20	20	LSCC	Plasma	qRT‐PCR	0.85	0.95	0.97	healthy controls
Han J(f) et al	2021	China	Hsa_circ_0122790	Upregulated	100	100	LSCC	Plasma	qRT‐PCR	0.83	0.95	0.91	healthy controls
Guo Y(a) et al	2020	China	CircMORC3	Downregulated	33	33	HPSCC	Tissue	qRT‐PCR	0.81	0.69	0.83	adjacent normal tissues
Guo Y(b) et al	2020	China	CircMORC3	Downregulated	22	22	HPSCC	Plasma	qRT‐PCR	0.72	0.68	0.77	vocal cord polyps tissues
Shen Z et al	2021	China	Hsa_circ_0016148	Downregulated	137	137	HNSCC	Tissue	qRT‐PCR	0.92	0.87	0.91	adjacent normal tissues
Sun S et al	2018	China	Hsa_Circ_001242	Downregulated	40	40	OSCC	Tissue	qRT‐PCR	0.73	0.78	0.78	adjacent normal tissues
Li B et al	2018	China	Hsa_Circ_0008309	Downregulated	45	45	OSCC	Tissue	qRT‐PCR	0.51	0.91	0.76	adjacent normal tissues
He T et al	2018	China	CircPVT1	Upregulated	50	50	OSCC	Tissue	qRT‐PCR	0.69	0.86	0.79	adjacent normal tissues
Zhao S et al	2018	China	Hsa_circ_0001874+ Hsa_circ_0001971	Upregulated	93	85	OSCC	Saliva	qRT‐PCR	0.93	0.78	0.92	healthy controls
Li X et al	2019	China	Hsa_circ_0004491	Downregulated	40	40	OSCC	Tissue	qRT‐PCR	0.73	0.68	0.75	adjacent normal tissues
Xia B et al	2019	China	CircMMP9	Upregulated	25	16	OSCC	Plasma	qRT‐PCR	0.89	0.81	0.91	healthy controls
Su W et al	2019	China	Hsa_circ_0005379	Downregulated	37	37	OSCC	Tissue	qRT‐PCR	0.70	0.61	0.68	adjacent normal tissues
Dou Z et al	2019	China	Hsa_circ_0072387	Downregulated	63	63	OSCC	Tissue	qRT‐PCR	0.71	0.70	0.75	adjacent normal tissues
Fan C(b) et al	2019	China	CircMAN1A2	Upregulated	55	121	OSCC	Serum	qRT‐PCR	0.67	0.92	0.78	healthy controls
Wang Z et al	2019	China	Hsa_circ_009755	Downregulated	27	27	OSCC	Tissue	qRT‐PCR	0.70	0.78	0.78	adjacent normal tissues
Zhang H et al	2020	China	Hsa_circ_0003829	Downregulated	60	60	OSCC	Tissue	qRT‐PCR	0.70	0.80	0.81	adjacent normal tissues
Li L et al	2020	China	Hsa_circ_0086414	Downregulated	55	55	OSCC	Tissue	qRT‐PCR	0.66	0.87	0.75	adjacent normal tissues
Chen G et al	2020	China	CircATRNL1	Downregulated	48	48	OSCC	Tissue	qRT‐PCR	0.85	0.51	0.71	adjacent normal tissues
Zhang B et al	2020	China	Hsa_circ_009755	Downregulated	42	42	OSCC	Tissue	qRT‐PCR	0.69	0.89	0.83	adjacent normal tissues
Fan X et al	2021	China	CircSPATA6	Downregulated	46	25	OSCC	Serum	qRT‐PCR	0.79	0.69	0.77	healthy controls

Abbreviations: AUC, area under the curve; HNSCC, head and neck squamous cell carcinoma; HPSCC, hypopharyngeal squamous cell carcinoma; LSCC, laryngeal squamous cell carcinoma; NPC, nasopharyngeal carcinoma; OSCC, oral squamous cell carcinoma; SEN, sensitivity; SPE, specificity.

**TABLE 2:** Main characteristics of studies for prognosis analysis in HNSCC

Study	Year	CircRNAs	Country	High	Low	Test method	Type	Sample type	Regulation pattern	Follow‐up months	Survival indicators
Shuai M et al	2018	Hsa_circ_0000285	China	105	45	qRT‐PCR	NPC	Serum	Upregulated	80	OS
Chen L et al	2019	CircRNA_000543	China	75	48	qRT‐PCR	NPC	Tissue	Upregulated	100	OS
Luo Y et al	2020	CircMYC	China	148	62	qRT‐PCR	NPC	Serum	Upregulated	60	OS, DFS
Shuai M et al	2020	Hsa_circ_001387	China	54	46	qRT‐PCR	NPC	Tissue	Upregulated	60	OS
Hong X et al	2021	CircCRIM1	China	91	127	qRT‐PCR	NPC	Tissue	Upregulated	120	OS, DFS
Ke Z et al	2020	CircHIPK3	China	32	31	qRT‐PCR	NPC	Tissue	Upregulated	150	OS
Dong Q et al	2020	Hsa_circ_0028007	China	160	81	qRT‐PCR	NPC	Tissue	Upregulated	40	OS
Fang X et al	2021	CircTRAF3	China	50	50	qRT‐PCR	NPC	Tissue	Upregulated	100	OS, DFS
Li W et al	2021	CircTGFBR2	China	29	46	ISH	NPC	Tissue	Downregulated	100	OS
Liu Z et al	2021	CircZNF609	China	35	25	qRT‐PCR	NPC	Tissue	Upregulated	60	OS
Verduci L et al	2017	CircPVT1	Italy	71	35	qRT‐PCR	HNSCC	Tissue	Upregulated	70	OS
Ju H et al	2021	CircGNG7	China	22	43	ISH	HNSCC	Tissue	Downregulated	60	OS
Zhang S et al	2021	Hsa_circ_0032822	China	30	30	qRT‐PCR	HNSCC	Tissue	Upregulated	120	OS, DFS
Shen Z et al	2021	Hsa_circ_0016148	China	65	72	qRT‐PCR	HNSCC	Tissue	Downregulated	60	OS
Wang Z et al	2020	CircMATR3	China	24	26	qRT‐PCR	HPSCC	Tissue	Upregulated	60	OS
Wu P et al	2021	CircCUX1	China	45	33	qRT‐PCR	HPSCC	Tissue	Upregulated	48	OS, DFS
Yao Y et al	2020	Hsa_circ_0001742	China	73	73	qRT‐PCR	TSCC	Tissue	Upregulated	60	OS
Qian C et al	2021	Hsa_circ_0043265	China	20	20	qRT‐PCR	TSCC	Tissue	Downregulated	60	OS
Qian C et al	2021	Hsa_circ_0000003	China	20	20	qRT‐PCR	TSCC	Tissue	Upregulated	60	OS
Wei Z et al	2019	Hsa_circ_0042666	China	18	17	qRT‐PCR	LSCC	Tissue	Downregulated	150	OS
Wang J et al	2019	CircFLNA	China	19	20	qRT‐PCR	LSCC	Tissue	Upregulated	200	OS
Gao W et al	2020	CircPARD3	China	50	50	qRT‐PCR	LSCC	Tissue	Upregulated	70	OS
Wang X et al	2020	Hsa_circ_103862	China	80	72	ISH	LSCC	Tissue	Upregulated	60	OS
Zang Y et al	2020	CircCCND1	China	50	51	qRT‐PCR	LSCC	Tissue	Upregulated	80	OS
Chu Y et al	2020	Hsa_circ_0067934	China	20	20	qRT‐PCR	LSCC	Tissue	Upregulated	90	OS
Wu Y et al	2021	circCORO1C	China	48	48	qRT‐PCR	LSCC	Tissue	Upregulated	70	OS
Dou Z et al	2019	Hsa_circ_0072387	China	15	63	qRT‐PCR	OSCC	Tissue	Downregulated	60	OS
Xia B et al	2019	CircMMP9	China	37	37	qRT‐PCR	OSCC	Tissue	Upregulated	80	OS
Hao C et al	2020	CircITCH	China	46	57	qRT‐PCR	OSCC	Tissue	Downregulated	60	OS
Li K et al	2020	Hsa_circ_0000745	China	32	32	qRT‐PCR	OSCC	Tissue	Upregulated	60	OS
Wang J et al	2020	CircEPSTI1	China	72	82	qRT‐PCR	OSCC	Tissue	Upregulated	60	OS
Luo Y et al	2020	Hsa_circ_0000199	China	68	40	qRT‐PCR	OSCC	Serum	Upregulated	60	OS, DFS
Yang Y et al	2020	Hsa_circ_002178	China	25	25	qRT‐PCR	OSCC	Tissue	Upregulated	60	OS
Peng Q et al	2020	Hsa_circ_0000140	China	28	28	qRT‐PCR	OSCC	Tissue	Downregulated	50	OS
Zhao W et al	2020	CircUHRF1	China	10	10	qRT‐PCR	OSCC	Tissue	Upregulated	50	OS
Gao L et al	2020	Hsa_circ_0092125	China	50	36	qRT‐PCR	OSCC	Tissue	Upregulated	60	OS
Chen H et al	2021	CircVAPA	China	30	30	qRT‐PCR	OSCC	Tissue	Upregulated	50	OS, DFS
Liu J et al	2021	CircIGHG	China	64	105	qRT‐PCR	OSCC	Tissue	Upregulated	40	OS

Abbreviations: DFS, disease‐free interval; HNSCC, head and neck squamous cell carcinoma; HPSCC, hypopharyngeal squamous cell carcinoma; ISH, in situ hybridization; LSCC, laryngeal squamous cell carcinoma; NPC, nasopharyngeal carcinoma; OS, overall survival; OSCC, oral squamous cell carcinoma; TSCC, tongue squamous cell carcinoma.

### 3.3 Expression of circRNAs with diagnosis in HNSCC

#### 3.3.1 Data analysis

**FIGURE 2:**
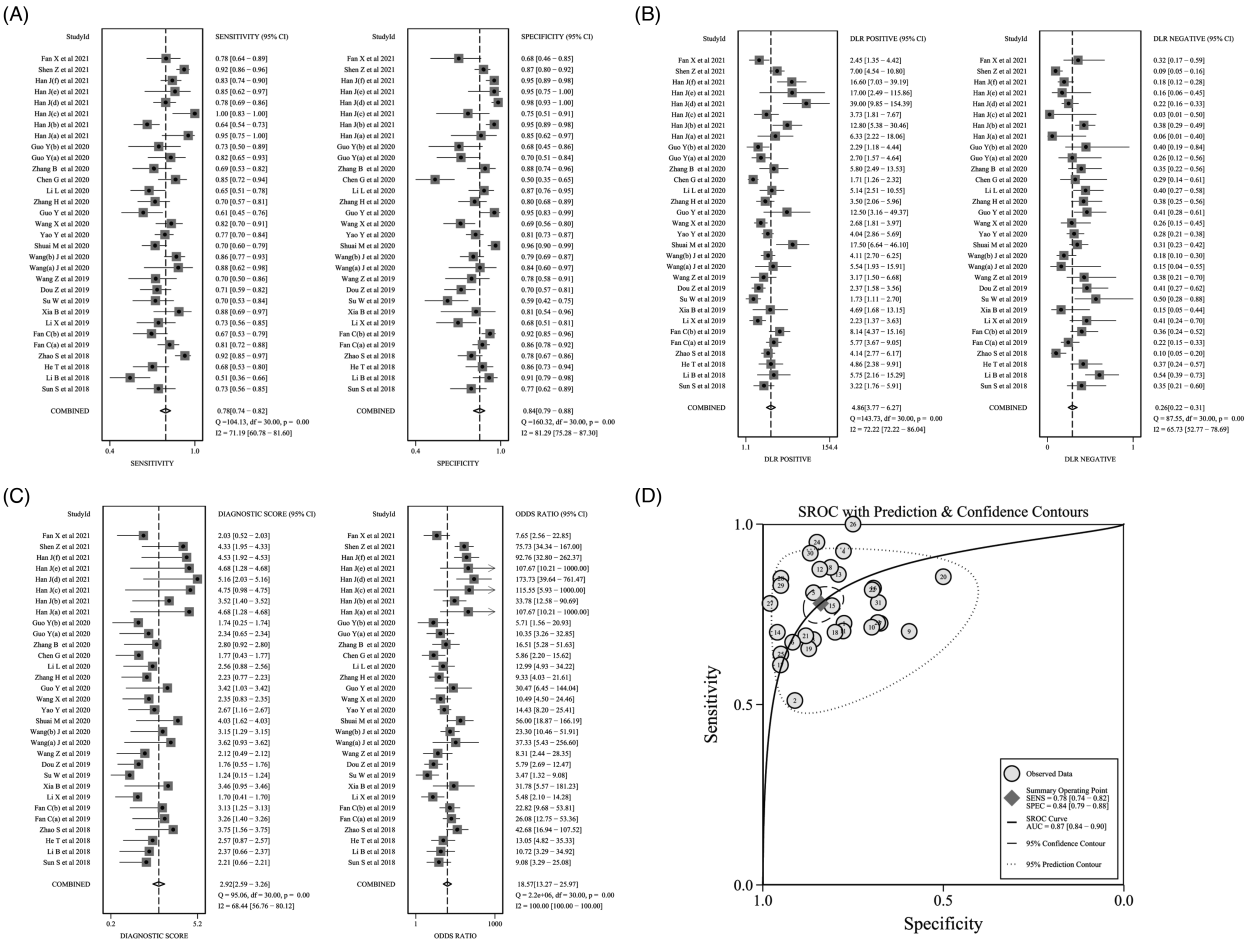
Forest plots of sensitivity, specificity, PLR, NLR, DOR, and AUC for diagnosis of circRNAs in HNSCC among 31 studies. (A) Sensitivity and specificity; (B) PLR and NLR; (C) DOR; (D) AUC (SROC curve)

Thirty‐one relevant studies from 23 articles were included in the meta‐analysis. As shown in Figure 2, there was significant heterogeneity in the pooled sensitivity (I^2^=71.19%, *p* < 0.001) and specificity (I^2^=81.29%, *p* < 0.001) values. Therefore, the random‐effects model was used to analyze diagnostic parameters. The forest diagram shows the value of circRNAs in the diagnosis of HNSCC; the pooled sensitivity was 0.78 (95% CI=0.74–0.82), specificity was 0.84 (95% CI=0.79–0.88), PLR was 4.86 (95% CI=3.77–6.27), NLR was 0.26 (95% CI=0.22–0.31), and the combined DOR was 19 (95% CI=13–26) (Figure 2A,B,C). In addition, Figure 2D shows a summary receiver operator characteristic (SROC) curve with an AUC of 0.87 (95% CI=0.84–0.90).

#### 3.3.2 Threshold effect, heterogeneity, and subgroup analysis

The Spearman’s correlation coefficient value was 0.313, and the *p* value was 0.086, indicating that the threshold effect was not observed. Figure 2D shows that there was no typical shoulder and arm, indicating that there was no threshold effect. This can also be equated with the fact that the threshold effect is not a source of heterogeneity.

**FIGURE 3:**
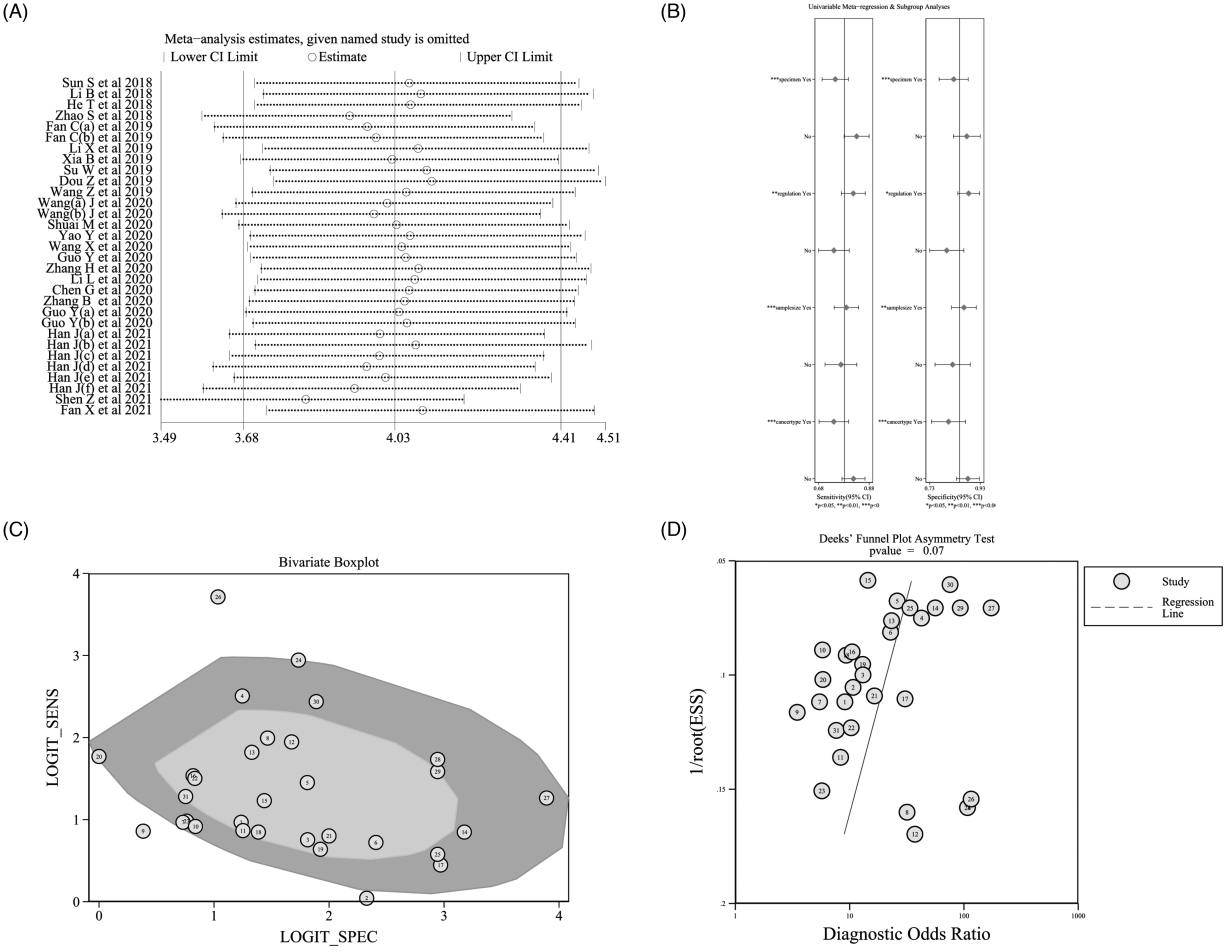
Assessment of the diagnostic accuracy of circRNAs in HNSCC. (A) Sensitivity analysis; (B) meta‐regression analysis; (C) bivariate boxplot; (D) Deek’s funnel plot

We have also shown the construction of a bivariate boxplot, which is a useful tool for detecting heterogeneity in each study (Figure 3C). Three studies did not occur in the boxplot, including studies 2, 20, and 26. Studies 26 involved the use of plasma, and studies 2 and 20 involved the use of tissue. This implies that the sample source could be the main cause of heterogeneity. Meta‐regression analysis showed that the sample size, specimen, circRNAs expression, and tumor type might decide the source of heterogeneity (Figure 3B).

Then, subgroup analysis was performed based on the circRNAs expression level, sample size, specimen, control source, and tumor type; results are shown in Table 3. The diagnostic performance of carcinogenic circRNAs was higher than that of tumor‐inhibiting circRNAs (AUC: 0.91 vs 0.82). The diagnostic performance of circRNAs in studies involving large samples was higher than that in studies involving small samples (AUC: 0.89 vs 0.84). With regard to the source of circRNAs extraction, plasma sample‐based studies exhibited the highest sensitivity (0.84), specificity (0.89), DOR (43), and AUC (0.92) values, compared with values in studies based on tissue or serum/saliva samples. In addition, circRNAs analysis was diagnostically effective for distinguishing patients with HNSCC from healthy individuals than for distinguishing HNSCC tissues from adjacent noncancerous tissues (AUC: 0.91 vs 0.83). Finally, the subgroup analysis of HNSCC based on tumor types from multiple parts indicated that circRNAs showed good diagnostic value for the detection of LSCC (AUC: 0.93), NPC (AUC: 0.90), and OSCC (AUC: 0.83). These results suggest that circRNAs may be an ideal diagnostic biomarker for HNSCC.

**TABLE 3:** Subgroup analysis of diagnostic accuracy of circRNAs for HNSCC

Variable	No	SEN (95% CI)	SPE (95% CI)	PLR (95% CI)	NLR (95% CI)	DOR (95% CI)	AUC (95% CI)	Heterogeneity	
								I^2^	*p*
Overall	31	0.78 (0.74–0.82)	0.84 (0.79–0.88)	4.9 (3.8–6.3)	0.26 (0.22–0.31)	19 (13–26)	0.87 (0.84–0.90)	98.0%	<0.001
Regulation									
Upregulated	16	0.81 (0.76–0.86)	0.88 (0.83–0.92)	6.7 (4.8–9.5)	0.21 (0.17–0.27)	32 (22–46)	0.91 (0.88–0.93)	96.0%	<0.001
Downregulated	15	0.74 (0.68–0.79)	0.78 (0.71–0.84)	3.4 (2.5–4.5)	0.33 (0.27–0.41)	10 ( 7–15)	0.82 (0.79–0.85)	93.0%	<0.001
Sample size									
>100	14	0.79 (0.73–0.83)	0.88 (0.82–0.92)	6.4 (4.4–9.3)	0.24 (0.19–0.30)	26 (17–42)	0.89 (0.86–0.92)	96.0%	<0.001
<100	17	0.76 (0.70–0.81)	0.79 (0.72–0.84)	3.6 (2.7–4.7)	0.30 (0.24–0.38)	12 ( 8–17)	0.84 (0.80–0.87)	94.0%	<0.001
Specimen									
Tissue	18	0.74 (0.69–0.79)	0.81 (0.75–0.86)	4.0 (3.0–5.3)	0.31 (0.26–0.38)	13 ( 9–18)	0.84 (0.80–0.87)	96.0%	<0.001
Plasma	9	0.84 (0.76–0.89)	0.89 (0.81–0.94)	7.8 (4.4–13.8)	0.18 (0.12–0.27)	43 (23–81)	0.92 (0.89–0.94)	93.0%	<0.001
Serum/Saliva	4	0.82 (0.71–0.89)	0.84 (0.77–0.90)	5.3 (3.7–7.5)	0.21 (0.14–0.34)	25 (16–39)	0.90 (0.87–0.92)	84.0%	=0.001
Source of control									
Adjacent	17	0.74 (0.69–0.79)	0.81 (0.74–0.86)	3.9 (2.9 –5.3)	0.32 (0.27–0.39)	12 (8–18)	0.83 (0.80–0.86)	96.0%	<0.001
Healthy/other	14	0.83 (0.78–0.88)	0.87 (0.81–0.91)	6.4 (4.4 –9.4)	0.19 (0.15–0.25)	33 (22–52)	0.91 (0.89–0.93)	95.0%	<0.001
Cancer type									
OSCC	15	0.74 (0.68–0.79)	0.79 (0.68–0.79)	3.5 (2.7 –4.5)	0.33 (0.27–0.40)	11 (7–15)	0.83 (0.79–0.86)	95.0%	<0.001
LSCC	8	0.82 (0.71–0.89)	0.92 (0.83–0.96)	9.8 (5.0 –19.3)	0.20 (0.13–0.32)	49 (24 –101)	0.93 (0.90–0.95)	94.0%	<0.001
NPC	4	0.81 (0.72–0.87)	0.88 (0.78–0.94)	6.6 (3.7 –11.6)	0.22 (0.16–0.31)	30 (18–51)	0.90 (0.87–0.92)	83.0%	=0.002
Other	4	0.82 (0.71–0.89)	0.80 (0.73–0.86)	4.1 (2.7 –6.3)	0.22 (0.13–0.39)	18 (7–47)	0.87 (0.84–0.90)	0	=0.336

Abbreviations: AUC, area under the curve; CI, confidence interval; DOR, diagnostic odds ratio; HNSCC, head and neck squamous cell carcinoma; LSCC, laryngeal squamous cell carcinoma; NLR, negative likelihood ratio; NPC, nasopharyngeal carcinoma; OSCC, oral squamous cell carcinoma; PLR, positive likelihood ratio; SEN, sensitivity; SPE, specificity.

#### 3.3.3 Publication bias and sensitivity analysis

Sensitivity analysis showed that the results of the meta‐analysis did not change when studies were omitted item by item (Figure 3A). The Deek’s funnel plot asymmetry test is a useful tool for assessing the potential publication bias in studies. The results of the use of this test showed that there was no significant publication bias, and the *p* value was 0.07 (Figure 3D).

### 3.4 Expression of circRNAs with prognosis in HNSCC

#### 3.4.1 Data analysis

**FIGURE 4:**
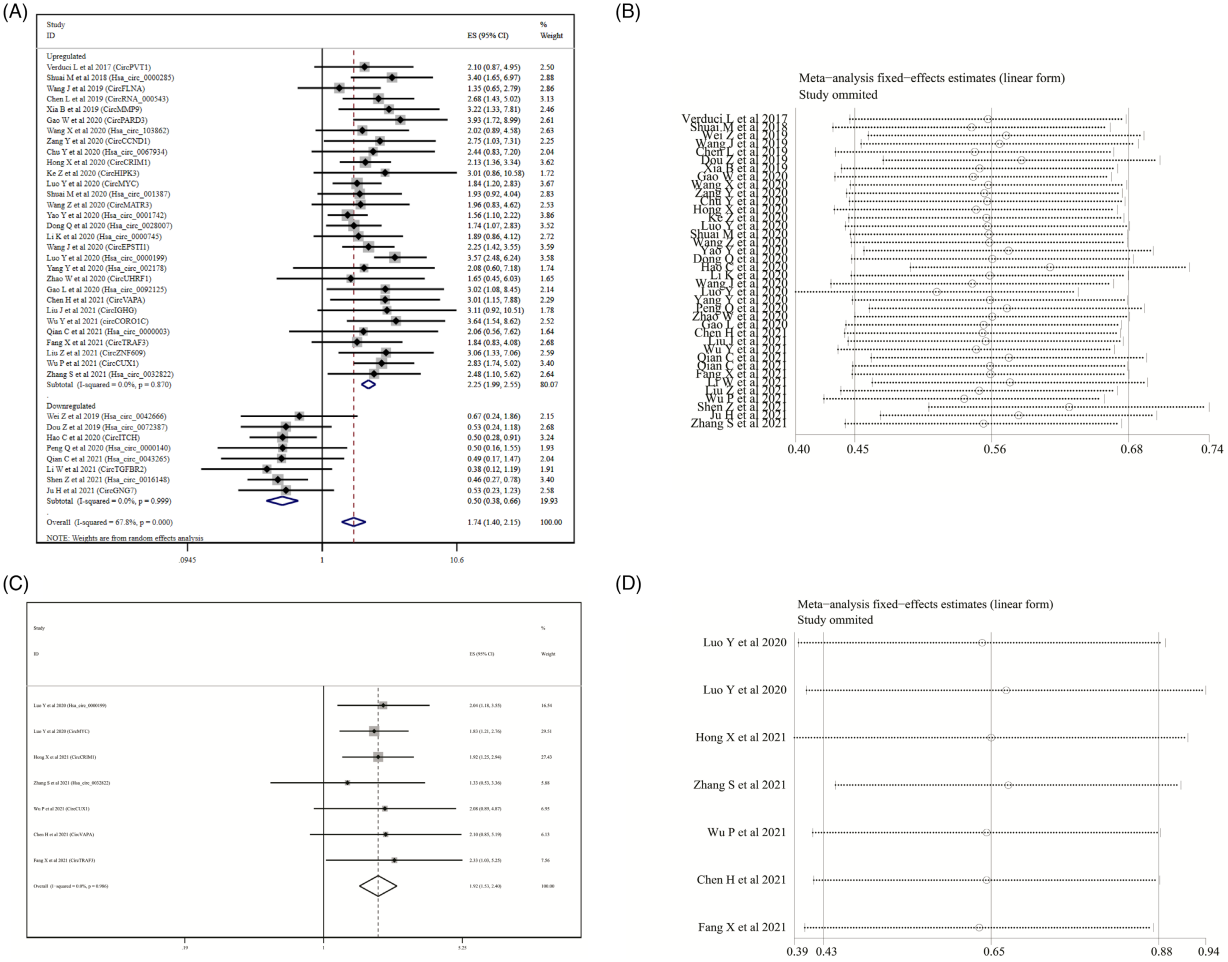
Forest plots and sensitivity analysis of the association between the expression of circRNAs and the prognosis of patients with HNSCC. (A) Forest plots for OS; (B) sensitivity analysis for OS; (C) forest plots for DFS; (D) sensitivity analysis for DFS

Survival analysis showed that oncogenic circRNAs overexpression was significantly associated with a worsened OS (HR=2.25, 95% CI: 1.99–2.55) and DFS (HR=1.92, 95%CI: 1.53–2.40), as shown in Figure 4A and 4C, respectively. In addition, the increased expression of tumor‐inhibiting circRNAs caused a prediction of improved OS (HR=0.50, 95%CI: 0.38–0.66), as shown in Figure 4A. These studies were all fixed‐effect models without significant heterogeneity.

#### 3.4.2 Heterogeneity and subgroup analysis

Subgroup analysis was further conducted according to the sample size, sample source, circRNAs detection method, and tumor type, to explore the source of heterogeneity. The results are shown in Table 4. The prognostic significance of upregulated circRNAs in OS was evaluated in 30 studies with 3058 HNSCC patients and its pooled HR was 2.25 (95%CI=1.99–2.55, I^2^=0.0, P_het_=0.870, fixed‐effects model), it suggested that the HNSCC patients with higher expression of circRNAs had shorter overall survival time than those with lower expression of circRNAs among tumor‐oncogene circRNAs, and among the downregulated circRNAs, the pooled HR for OS was 0.50 (95%CI=0.38‐0.66, I^2^=0.0, P_het_=0.999, fixed‐effects model), and it suggested that the higher expression of tumor suppressor circRNAs in HNSCC was associated with longer overall survival time. In terms of tumor type, a high level of circRNAs expression was associated with OSCC (OS: HR=1.70; 95%Cl=1.06–2.73), LSCC (OS: HR=2.12; 95%Cl=1.52–2.96), NPC (OS: HR=2.04; 95%Cl=1.67–2.48), and HPSCC (OS: HR=2.56; 95%Cl=1.63–4.01) and was associated with a poor prognosis. Though a high level of expression of circRNAs was associated with TSCC (OS: HR=1.22; 95%Cl=0.58–2.55), no defined correlation was observed. Only seven studies were related to DFS and tumor‐oncogene circRNAs, so we were unable to conduct further analysis.

**TABLE 4:** Subgroup analysis of prognostic outcomes of circRNAs for HNSCC

Variable	No	Patients	HR (95%CI)	*p*‐value	Heterogeneity		
					I^2^ (%)	*P*_*HET*_	Model
Overall Survival							
Overall	38	3647	1.74 (1.40–2.15)	<0.001	67.8	<0.001	Random
Regulation							
Upregulated	30	3058	2.25 (1.99–2.55)	<0.001	0	0.870	Fixed
Downregulated	8	589	0.50 (0.38–0.66)	<0.001	0	0.999	Fixed
Sample size							
>100	14	2118	1.89 (1.37– 2.60)	<0.001	78.1	0.2908	Random
<100	24	1529	1.62 (1.21–2.16)	0.005	56.8	0.2736	Random
Specimen							
Tissue	35	3179	1.65 (1.32–2.06)	<0.001	66.4	<0.001	Random
Serum	3	468	2.73 (1.71–4.35)	<0.001	58.7	0.089	Random
Test method							
qRT‐PCR	35	3355	1.84 (1.49–2.28)	<0.001	65.9	<0.001	Random
ISH	3	292	0.77 (0.28–2.16)	0.625	73.0	0.025	Random
Cancer type							
OSCC	12	1022	1.70 (1.06–2.73)	0.028	76.1	<0.001	Random
LSCC	7	563	2.12 (1.52–2.96)	<0.001	42.0	0.111	Fixed
NPC	10	1340	2.04 (1.67–2.48)	<0.001	30.4	0.166	Fixed
HNSCC	4	368	1.03 (0.42–2.52)	0.947	82.5	0.001	Random
TSCC	3	226	1.22 (0.58–2.55)	0.599	53.7	0.115	Random
HPSCC	2	128	2.56 (1.63–4.01)	<0.001	0	0.475	Fixed
Disease‐free survival							
Overall (upregulated)	7	834	1.92 (1.53–2.4)	<0.001	0	0.986	Fixed

Abbreviations: CI, confidence interval; Fixed, fixed‐effects model; HNSCC, head and neck squamous cell carcinoma; HPSCC, hypopharyngeal squamous cell carcinoma; HR, hazard ratio; LSCC, laryngeal squamous cell carcinoma; NPC, nasopharyngeal carcinoma; OSCC, oral squamous cell carcinoma; P_het_, *p* value of heterogeneity; Random, random‐effects model; TSCC, tongue squamous cell carcinoma.

#### 3.4.3 Publication bias and sensitivity analysis

**FIGURE 5:**
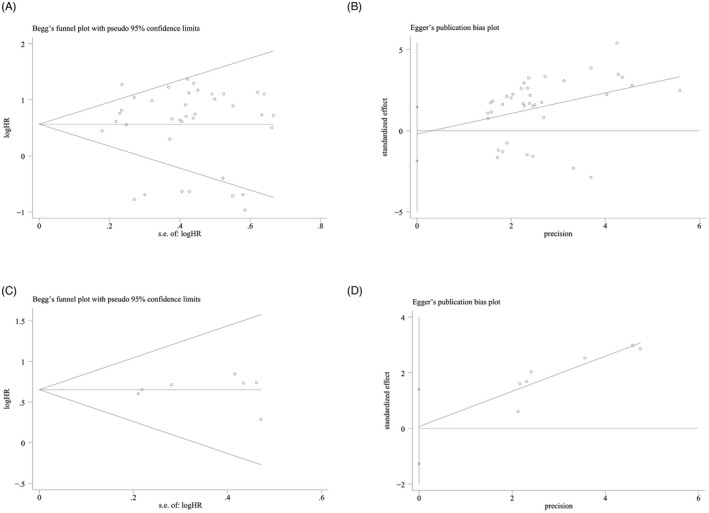
Publication bias of the association between the expression of circRNAs and the prognosis of patients with HNSCC. (A) Begg’s funnel plot for OS; (B) Egger's test plot for OS; (C) Begg’s funnel plot for DFS; (D) Egger’s test plot for DFS

The results of sensitivity analysis showed that no single study could affect the combined HRs of the OS and DFS (Figure 4B, D). To track the potential publication bias during the meta‐analysis, we conducted certain tests (Figure 5A,B,C,D). The *p* values of Begg's and Egger's tests for the OS and DFS were all greater than 0.05, indicating that there was no publication offset.

### 3.5 Expression of circRNA with clinicopathological parameters in HNSCC

In 27 included studies on clinicopathological parameters from 26 articles, a total of 27 circRNAs were described. The correlation between circRNAs and clinicopathological parameters of HNSCC patients is shown in Table 5. Our results showed that among these the clinicopathological features, oncogenic circRNA upregulation was associated with tumor size (OR=3.59, 95%CI=2.48–5.19, *p* < 0.001), degree of differentiation (OR=1.89, 95%CI=1.36–2.61, *p* < 0.001), TNM staging (OR=2.35, 95%CI=1.94–2.85, *p* < 0.001), lymph node metastasis (OR=1.85, 95%CI=1.23–2.78, *p* = 0.003), and distant metastasis (OR=3.42, 95%CI=2.42–4.84, *p* < 0.001). The upregulation of circRNA was associated with an improvement in clinicopathological features and lymph node metastasis (OR=0.25, 95%CI=0.14–0.47, *p* < 0.001). There was no statistical correlation between the expression of tumor suppressor gene circRNAs and age, sex, tumor size, tumor stage, and differentiation.

**TABLE 5:** Association between expression of circRNAs and clinicopathological features

Categories	Tumor promoter							Tumor suppressor						
	Studies	Patients	OR (95% CI)	*p*	I^2^	*P*_*HET*_	Model	Studies	Patients	OR (95% CI)	*p*	I^2^	*p*_*het*_	Model
Age (old/young)	20	1864	1.15 (0.95–1.40)	0.141	0.0%	0.636	Fixed	4	234	0.80 (0.48–1.35)	0.408	0.0%	0.487	Fixed
Gender (M/W)	22	2182	1.03 (0.85–1.24)	0.772	0.0%	0.949	Fixed	4	234	1.01 (0.60–1.70)	0.983	0.0%	0.905	Fixed
Tumor size (large/small)	8	550	3.59 (2.48–5.19)	<0.000	0.0%	0.939	Fixed	1	40	0.44 (0.12–1.57)	0.207	–	–	–
Differentiation grade (poor/well)	9	1052	1.89 (1.36–2.61)	<0.000	0.0%	0.872	Fixed	1	56	0.38 (0.12–1.19)	0.098	–	–	–
TNM stage (III+IV/I+II)	20	1952	2.35 (1.94–2.85)	<0.000	55.2%	0.002	Random	3	131	0.87 (0.12–6.57)	0.895	86.1%	0.001	Random
T classification (T3 + T4/T1 + T2)	13	1453	1.53 (0.94–2.47)	0.085	76.4%	<0.000	Random	–	–	–	–	–	–	–
Lymph node metastasis (Y/N)	15	1500	1.85 (1.23–2.78)	0.003	67.7%	<0.000	Random	3	194	0.25 (0.14–0.47)	<0.000	49.4%	0.139	Fixed
Distant metastasis (Y/N)	7	659	3.42 (2.42–4.84)	<0.000	10.6%	= 0.348	Fixed	1	103	0.39 (0.17–0.91)	0.029	–	–	–

Abbreviations: CI, confidence interval; Fixed, fixed‐effects model; Random, random‐effects model; OR, odds ratio; P_het_, *p* value of heterogeneity.

## 4 DISCUSSION

CircRNAs seem to have good prospects as an ideal biomarker for human cancer diagnosis or prognosis over the last decade due to their special advantages as a biomarker, which include their stable and continuous covalent closed loops, high stability in cells and body fluids, and close association between its complex biological functions and carcinogenesis^20^.

Four previous meta‐analyses by Wang^80^, Ding^81^, Li^82^, and Tan^83^ examined the association between circRNAs and cancer, and confirmed that circRNAs might play an important role in the diagnosis and prognosis of human cancer. The predictive role of circRNAs in different malignancies, including esophageal cancer^84^, lung cancer^85^, and colorectal cancer^86^, has also been confirmed recently. However, there are few articles on HNSCC tumors in these meta‐analyses. A growing number of studies have shown that some circRNAs are abnormally expressed in HNSCC^32^, ^37^, ^39^, ^65^, ^66^, ^67^, ^68^. However, the predictive value of circRNAs in HNSCC is still unclear. To our knowledge, this is the first meta‐analysis to address the relationship between the expression of circRNAs and the diagnosis, prognosis, and clinicopathological features of HNSCC.

In our analysis, overall, the pooled sensitivity and specificity of circRNAs for the diagnosis of HNSCC were 0.78 and 0.84, respectively, and the AUC was 0.87. Furthermore, the overall DOR was 19, while the combined PLR and NLR were 4.86 and 0.26, respectively. In addition, through subgroup analysis, we found that circRNAs were effective for the diagnosis of different HNSCC tumor types, especially LSCC (AUC: 0.93), NPC (AUC: 0.90), and OSCC (AUC: 0.83). The SROC curve and Spearman correlation coefficient indicated that there were no threshold effects. This indicates that the threshold effect is not the source of heterogeneity. Considering the significant heterogeneity, we chose the random‐effects model. However, the results of the bivariate boxplot and meta‐regression analysis indicate that the sources of heterogeneity between the included studies may be the sample size, tumor type, circRNAs expression, control source, and sample. These results suggest that circRNAs may be suitable for use as potential biomarkers for the diagnosis of HNSCC.

To determine the relationship between circRNA, OS, and DFS in HNSCC patients, a total of 38 eligible prognostic studies were included. Overall, the high expression of oncogenic RNA resulted in a significant deterioration in the OS, whereas the high expression of inhibited circRNAs resulted in a significantly better OS in HNSCC patients. In addition, when grouped by tumor types, the high expression of circRNAs was indicative of a worsened prognosis for patients with OSCC, LSCC, NPC, and HPSCC, but not those with TSCC. This may be attributable to the limited number of studies on individuals with TSCC (*n* = 3). During our search, seven studies examined the association of circRNAs with DFS, and we found that the overexpression of oncogenic circRNAs was associated with a shorter DFS.

The upregulation of circRNAs was significantly correlated with the tumor size, degree of differentiation, TNM stage, lymph node metastasis, and distant metastasis. The downregulation of circRNAs, a tumor suppressor gene, led to poor lymph node metastasis.

However, certain limitations are associated with our meta‐analysis. First, most of the demographic data included in the meta‐analysis were from China; hence, our conclusions were more applicable to the Chinese or Asian population, which may affect the applicability of our findings across different regions. Second, the number of included studies on circRNA, a tumor suppressor gene, is relatively small, and more studies need to be conducted in the future, to further confirm the results. In addition, some studies do not clearly state the sensitivity, specificity, or HR values. We extracted essential data from the ROC and KM curves provided, which could lead to potential deviations. Finally, although we performed a hierarchical analysis, heterogeneity was still observed in some subgroups.

## 5 CONCLUSION

Taken together, our meta‐analysis showed that circRNAs can be used as promising biomarkers for the diagnosis of patients with HNSCC, and especially for those with LSCC and NPC. Furthermore, our study also found that there is a significant association between circRNAs overexpression, prognostic outcomes, and clinicopathological values in patients with HNSCC. This implies that circRNAs might play an important role in the occurrence and development of HNSCC. However, more comprehensive, high‐quality, and large‐scale studies involving populations from more regions need to be performed, to elucidate the roles of circRNAs in HNSCC.

## 7 CONFLICT OF INTEREST

None declared.

8

## Supporting information


Figure S1

## Data Availability

The original contributions presented in the study are included in the article/supplementary material; further reasonable inquiries can be directed to the corresponding author/s.
