# Supplementary figures and images for: Diagnostic and prognostic value of circRNAs expression in head and neck squamous cell carcinoma: A meta‐analysis
Source: J Clin Lab Anal. 2022 May 20;36(7):e24496. doi: 10.1002/jcla.24496 (PMC9280010; doi:10.1002/jcla.24496)

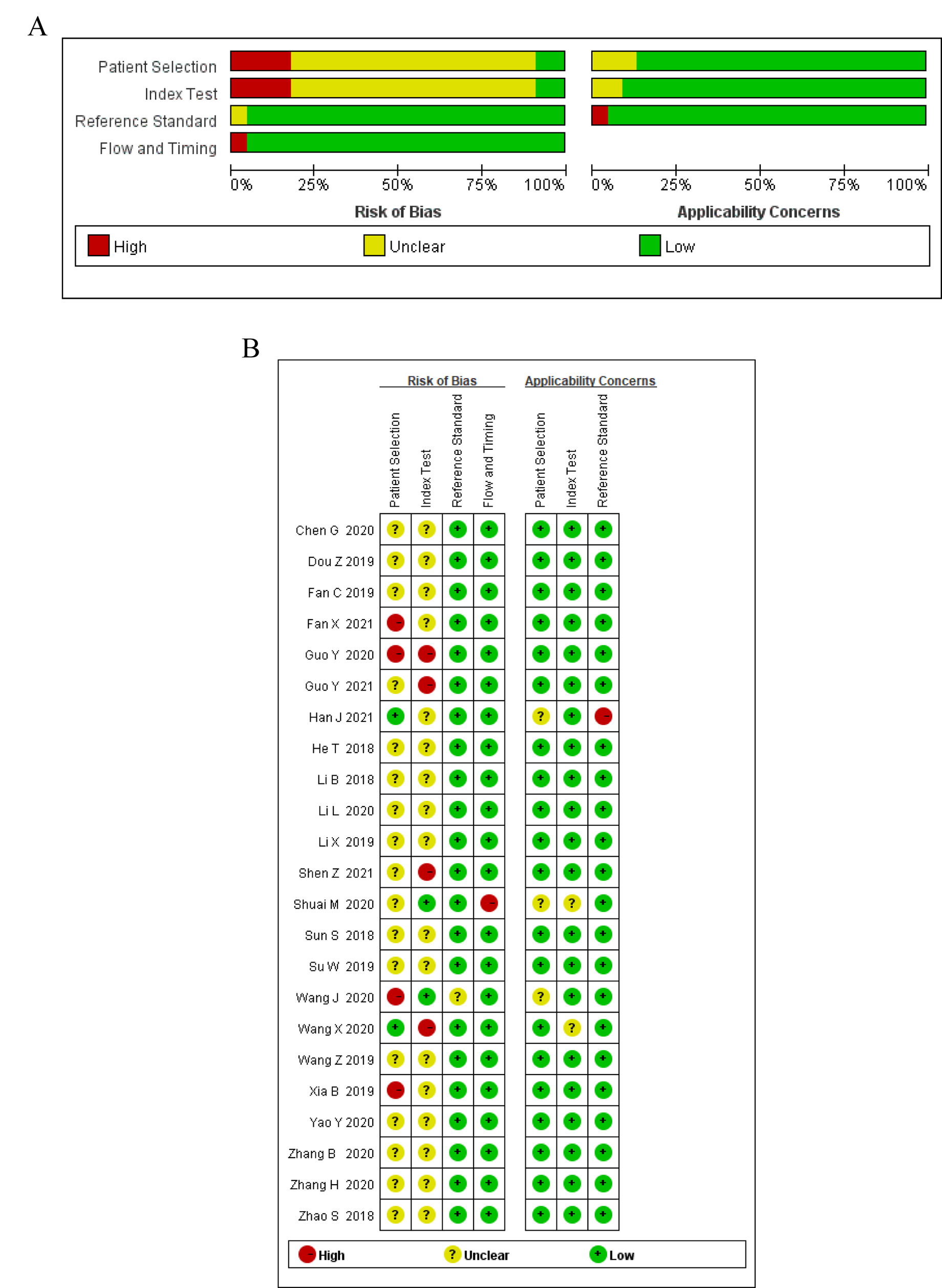

Supplement: Supplementary file 1 — Figure S1 [file JCLA-36-e24496-s001.tif]
